# Data quality in diffusion tensor imaging studies of the preterm brain: a systematic review

**DOI:** 10.1007/s00247-015-3307-y

**Published:** 2015-03-29

**Authors:** Kay Pieterman, Annemarie Plaisier, Paul Govaert, Alexander Leemans, Maarten H. Lequin, Jeroen Dudink

**Affiliations:** 1 Division of Neonatology, Department of Pediatrics, Erasmus Medical Center – Sophia, dr. Molewaterplein 60, 3015 GJ Rotterdam, The Netherlands; 2 Department of Radiology, Erasmus Medical Center, Rotterdam, The Netherlands; 3 Department of Pediatrics, Koningin Paola Children’s Hospital, Antwerp, Belgium; 4 Image Sciences Institute, University Medical Center Utrecht, Utrecht, The Netherlands

**Keywords:** Diffusion tensor imaging, Image acquisition, Neonate, Prematurity, Magnetic resonance imaging, Systematic review

## Abstract

**Background:**

To study early neurodevelopment in preterm infants, evaluation of brain maturation and injury is increasingly performed using diffusion tensor imaging, for which the reliability of underlying data is paramount.

**Objective:**

To review the literature to evaluate acquisition and processing methodology in diffusion tensor imaging studies of preterm infants.

**Materials and methods:**

We searched the Embase, Medline, Web of Science and Cochrane databases for relevant papers published between 2003 and 2013. The following keywords were included in our search: prematurity, neuroimaging, brain, and diffusion tensor imaging.

**Results:**

We found 74 diffusion tensor imaging studies in preterm infants meeting our inclusion criteria. There was wide variation in acquisition and processing methodology, and we found incomplete reporting of these settings. Nineteen studies (26%) reported the use of neonatal hardware. Data quality assessment was not reported in 13 (18%) studies. Artefacts-correction and data-exclusion was not reported in 33 (45%) and 18 (24%) studies, respectively. Tensor estimation algorithms were reported in 56 (76%) studies but were often suboptimal.

**Conclusion:**

Diffusion tensor imaging acquisition and processing settings are incompletely described in current literature, vary considerably, and frequently do not meet the highest standards.

**Electronic supplementary material:**

The online version of this article (doi:10.1007/s00247-015-3307-y) contains supplementary material, which is available to authorized users.

## Introduction

The rate of premature birth is rising globally [1]. Although improvements in obstetric and neonatal care have resulted in increased survival rates, neurodevelopmental outcome remains a source of concern because many preterm infants have neuromotor, cognitive and behavioral disabilities that persist in later life [2, 3]. White matter injury is suggested to account for many neurological sequelae among preterm infants, and although cystic periventricular leukomalacia is becoming less common, diffuse non-cystic white matter changes such as alterations in signal intensity and punctate white matter lesions are frequently observed [4–7]. Major changes of fetal white matter take place during the final stages of a normal pregnancy [8]. Infants born preterm undergo these changes in a high-risk extra-uterine environment, which poses risks for normal brain ontogenesis. Diffusion tensor imaging allows us to objectively assess these (microstructural) changes by mapping restricted random motion of water molecules within white matter tissue in vivo [9, 10].

Objective quantification of white matter microstructure and integrity using diffusion tensor imaging (DTI) may elucidate the impact of preterm birth and related sequelae on neurodevelopment, and DTI has the potential to provide early biomarkers of subsequent neurodevelopmental outcome [5, 7, 11–13]. Sophisticated applications of diffusion tensor imaging such as voxel-based analyses and fiber tractography enable visualization and quantification of specific white matter tracts in vivo. Several studies using these techniques have provided important insights into brain development and the impact of injury on functional outcome [14–18]. Recent projects to explore whole-brain connectivity are very promising because mapping neural circuits may help in the understanding of injury mechanisms responsible for neurocognitive impairment [19–21].

However, brain imaging in this specific vulnerable population is quite challenging. Obtaining good-quality data is complicated by the fact that diffusion tensor imaging is intrinsically highly sensitive to artefacts [22–24] and these infants tend to move more and have smaller head sizes and higher heart- and breathing rates than adults [25, 26]. The preterm infant population should be regarded as one of the most challenging patient groups to image using diffusion tensor imaging, and therefore requires maximal awareness of the acquisition and processing steps that determine data quality. Obtaining reliable diffusion tensor imaging data in this specific population can only be achieved when acquisition, quality assessment and data processing steps meet the highest standards possible.

Recently we demonstrated that good-quality diffusion tensor imaging (DTI) data and a well-informed choice of processing methodology have a serious influence on tract characteristics derived from neonatal DTI datasets [27]. Among others, different tensor estimation methods handle outliers and errors differently, and because datasets obtained from preterm infants generally contain a large number of artefacts, this kind of methodological considerations could have a major influence on study results.

The purpose of this study is to evaluate information obtained from diffusion tensor imaging studies of preterm infants, with a focus on acquisition settings, processing methodology and data quality assessment. Therefore, we conducted a systematic review of the literature.

## Materials and methods

The Embase, Medline, Web of Science and Cochrane database were systematically searched for relevant papers published between 2003 and September 2013 by two reviewers (K.P., A.P.), each with more than 3 years of experience in neonatal diffusion tensor imaging. The search was performed Oct. 5, 2013, and included synonyms and combinations of the following keywords: prematurity, neuroimaging, brain and diffusion tensor imaging. We included English-written studies in healthy and non-healthy infants. Non-human research, case reports, reviews and editorials were excluded. Studies were considered relevant when they met the following criteria: (1) they included preterm infants born at <32 weeks’ gestation, (2) MRI was performed within the first 28 days after term-equivalent age, and (3) diffusion tensor imaging was incorporated in study design and discussed in the results.

We extracted information regarding:

1. The use of a neonatal-specific head coil or an MRI-compatible neonatal incubator with a dedicated neonatal head coil, and the use of sedative drugs prior to diffusion tensor imaging acquisition.
2. Acquisition parameters with regard to diffusion tensor imaging analysis (magnetic field strength, number of gradient directions, b-value, number of non-diffusion-weighted images).
3. Processing methods (assessment of diffusion tensor imaging data quality, correction for motion and distortions, methods of diffusion tensor estimation and data analysis).

## Results

**Fig. 1:**
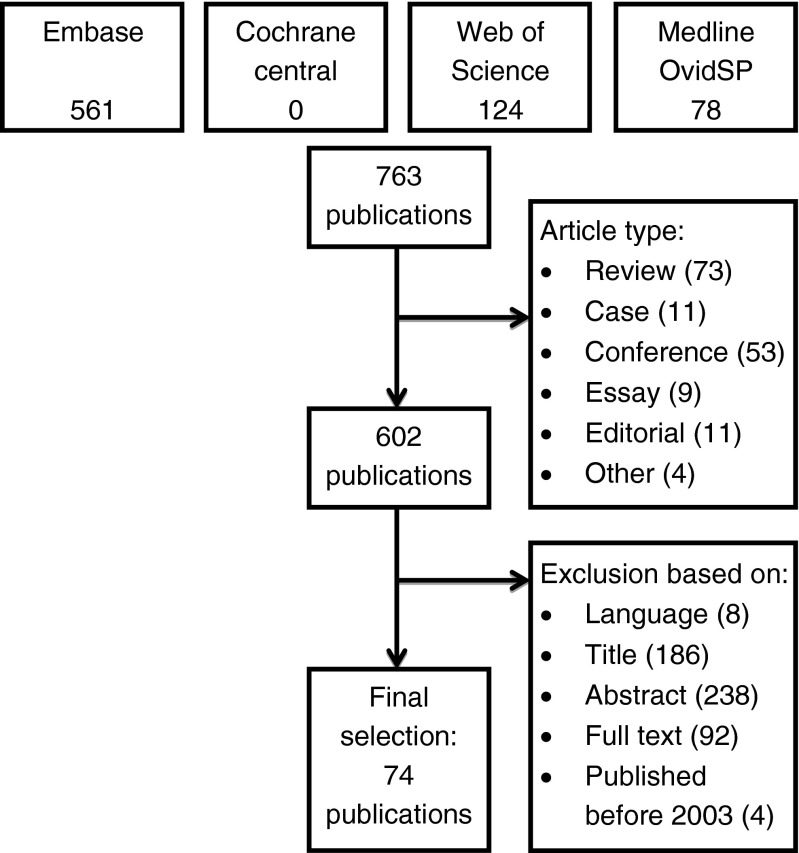
Flowchart of the literature search

The initial search resulted in 763 articles. All titles and abstracts were screened for relevance, after which the full text versions of 170 seemingly relevant articles were read. Seventy-four articles met our inclusion criteria (Fig. 1). A summary of these is given in Table 1.

**Table 1:** Details of diffusion tensor imaging studies included in review

Reference	Neo. coil	MRI incub	Sed	MRI (T)	No. dir	No. *b* = 0	b-value (s/mm^2^)	DTI analysis	Quality assess.	Quality method	Correction method	DTI excl	DTE method
Adams et al. 2010 [28]	+	+	-		12	1	600 700	ROI analysis Determ tract	+			+	WLLS
Aeby et al. 2012 [29]	-	-	-	1.5	32	1	600	ROI analysis	+	Screening	Motion	-	WLLS
Aeby et al. 2013 [30]	-	-	-	1.5	32	1	600	TBSS	+	Screening		+	WLLS
Als et al. 2004 [31]	-	-	-	1.5	6	1	750	ROI analysis	+	Screening		+	
Anjari et al. 2007 [32]	-	-	+	3.0	15	1	750	TBSS	-		Eddy currents	+	NLLS
Anjari et al. 2009 [33]	-	-	+	3.0	15	1	750	TBSS	+		Eddy currents	+	LLS
Arichi et al. 2010 [34]	-	-	+	3.0	32	1	750	Prob tract	+	Screening	Eddy currents	+	LLS
Arzoumanian et al. 2003 [35]	-	-	-	1.5	6	2	1,000	ROI analysis	+	Software	Eddy currents Motion	+	LLS
Ball et al. 2013 [36]	-	-	+	3.0	15	1	750	TBSS	+	Screening	Eddy currents Motion	-	LLS
Ball et al. 2013 [14]	-	-	+	3.0	15	1	7450	ROI analysis, TBSS	+	Screening	Eddy currents Motion	+	
Ball et al. 2010 [37]	-	-	+	3.0	15	1	750	TBSS	+	Screening	Eddy currents	-	LLS
Ball et al. 2012 [38]	-	-	+	3.0	15		750	TBSS	+	Screening		+	LLS
Ball et al. 2013 [14]	-	-	+	3.0	32	1	750	Prob tract	-		Eddy currents	-	LLS
Bassi et al. 2008 [15]	-	-	+	3.0	15	1	750	TBSS Prob tract	+		Eddy currents	+	LLS
Bassi et al. 2011 [11]	-	-	+	3.0	15	1	750	TBSS Prob tract	+		Eddy currents	-	LLS
Berman et al. 2005 [39]	+	+	-	1.5	6	1	600	Determ tract Prob tract	+	Screening	Motion	+	WLLS
Berman et al. 2009 [40]	+	+	-	1.5	6	1	600	Determ tract	-		Motion	-	WLLS
Bonifacio et al. 2010 [41]	+	+	+	1.5	12	1	600 700	ROI analysis	+	Screening		+	WLLS
Brummelte et al. 2012 [42]	+	+	-	1.5	12		600 700	ROI analysis	-			-	
Chau et al. 2009 [43]	-	-	-	1.5	12	1	600 700	ROI analysis	+		Eddy currents Motion	+	
Chau et al. 2012 [44]	+	+	-	1.5	12	1	600 700	ROI analysis	-		Eddy currents	-	WLLS
Cheong et al. 2009 [45]	-	-	-	1.5	6	2	700	ROI analysis	-			-	WLLS
Counsell et al. 2006 [46]	+	-	+	1.5	6		710	ROI analysis	+	Screening	Eddy currents	-	WLLS
de Bruine et al. 2011 [16]	-	-	+	3.0	32	1	1,000	Determ tract	+	Screening		+	LLS
de Bruine et al. 2013 [47]	-	-	+	3.0	32		1,000	Tractography	+	Screening		+	
Delpolyi et al. 2005 [48]	+	+	+	1.5	6	1	600	ROI analysis	+			+	WLLS
Drobyshevsky et al. 2007 [49]	-	-	-		6		1,000	ROI analysis	+	Screening		+	WLLS
Dudink et al. 2007 [50]	+	+	-	1.5	25		1,000	ROI analysis	+	Screening		+	LLS
Dudink et al. 2008 [51]	-	-	+	3.0	6	1	350 700 1,500 3,000	ROI analysis	-			+	
Dudink et al. 2010 [52]	+	+	-	1.5	25	1	1,000	ROI analysis	+	Screening		+	LLS
Gimenez et al. 2008 [53]	-	-	-	3.0	6	1	500 1,000	ROI analysis	+	Screening	Eddy currents Motion	+	WLLS
Glass et al. 2010 [54]	+	+	+	1.5	6	1	600	Prob tract	+		Motion	+	NLLS
Groppo et al. 2012 [55]	-	-	+	3.0	32	1	750	Prob tract	-		Eddy currents	-	LLS
Hasegawa et al. 2011 [56]	-	-	+	1.5	15	1	1,000	Determ tract	+			+	
Hemels et al. 2012 [57]	-	-	+	3.0				ROI analysis	+		Motion	+	
Jo et al. 2012 [58]	-	-	-	1.5	32	1	600	Prob tract	-		Eddy currents Motion	-	LLS
Lee et al. 2013 [59]	-	-	-	1.5	32		1,000	TBSS	-			-	NLLS
Lee et al. 2013 [60]	-	-	-	1.5	25	1	800	ROI analysis	+	Screening		+	LLS
Lepomaki 2012 [61]	-	-	-	1.5	15		600	ROI analysis	+	Screening		+	
Lepomaki et al. 2013 [62]	-	-	-	1.5	15		600 1200	TBSS	+	Screening	Eddy currents Motion	+	LLS
Lepomaki et al. 2012 [63]	-	-	-	1.5	15		600	ROI analysis	+	Screening		+	
Lepomaki et al. 2013 [64]	-	-	-	1.5	15	1	600 1200	TBSS	+	Screening	Eddy currents Motion	+	
Ling et al. 2013 [65]	-	-	+	1.5	15	1	1,000	ROI analysis	+	Screening	Yes, unknown	+	LLS
Liu et al. 2010 [66]	-	-	-	1.5	32		600	Prob tract	+	Screening	Eddy currents Motion	-	LLS
Liu et al. 2011 [67]	-	-	-	1.5	32	1	600	Prob tract	+	Defined	Eddy currents Motion	+	LLS
Liu et al. 2012 [68]	-	-	-	1.5	32		600	Prob tract	+	Screening	Eddy currents Motion	+	LLS
Maas et al. 2004 [69]	+	+	-	1.5	6	1	600	ROI analysis	+			-	WLLS
Mathew et al. 2013 [70]	+	+	-	3.0	30	1	1,000	ROI analysis	+	Screening	Motion, b-matrix, intensity inhomogeneity, susceptible distortions	+	LLS
Melbourne et al. 2012 [71]	-	-	-		30	2	600	Automated Segmentation	+	Screening		+	
Milgrom et al. 2010 [72]	-	-	-	1.5				ROI analysis	+	Screening		+	NLLS
Nijman et al. 2013 [73]	-	-	+	3.0	32	1	800	ROI analysis	-	Screening	Eddy currents Motion	+	
Nossin-Manor et al. 2013 [74]	+	+	-	1.5	15	3	700	ROI analysis	+	Software	Motion	+	NLLS/ LLS
Paquette et al. 2013 [75]	+	+	-	1.5	25		700	TBSS	+	Screening		+	LLS
Partridge et al. 2004 [76]	+	+	-	1.5	6	1	600	ROI analysis	+	Screening	Eddy currents	+	WLLS
Partridge et al. 2005 [77]	+	+	-	1.5	6	1	600	Determ tract	+	Screening	Eddy currents Motion	-	WLLS
Pogribna et al. 2013 [78]	-	-	-	3.0	15	1	800	ROI analysis	+	Screening		+	LLS
Pogribna et al. 2013 [79]	-	-	-	3.0	15	1	800	ROI analysis	+	Screening		+	WLLS
Ratnarajah et al. 2013 [80]	-	-	-	1.5	19	1	600	Atlas Tractography	+	Screening	Eddy currents Motion	+	WLLS
Reiman et al. 2009 [81]	-	-	-	1.5	15	1	600	ROI analysis	+	Screening		+	
Rogers et al. 2012 [82]	-	-	-	1.5	6	2	700	ROI analysis	-			-	LLS
Rose et al. 2008 [83]	-	-	-	1.5	44	16	1,100	TBSS	+	Screening	Eddy currents Motion	+	LLS
Rose et al. 2009 [84]	-	-	-	1.5	6	2	1,000	ROI analysis	+	Screening		+	WLLS
Shim et al. 2012 [85]	-	-	+	3.0	30	1	700	TBSS	+	Screening	Eddy currents Motion	+	LLS
Skiold et al. 2010 [86]	-	-	+	1.5	15	1	700	ROI analysis	+	Screening	Eddy currents Motion	+	WLLS
Tam et al. 2009 [87]	+	+	-		6	4	600	ROI analysis	-			-	
Thompson et al. 2011 [88]	-	-	-	1.5	6	2	700	Prob tract	+	Screening	Eddy currents Motion	+	LLS
Thompson et al. 2012 [89]	-	-	-	1.5	6	2	700	ROI analysis Prob tract	+	Screening	Eddy currents	+	WLLS
Tymofiyeva et al. 2013 [21]	-	-	-	3.0	30		700	Tractography Network analysis	+	Screening	Eddy currents Motion	+	WLLS
van Kooij et al. 2011 [90]	-	-	+	3.0	32	1	800	Determ tract	+	Screening		+	
van Kooij et al. 2012 [17]	-	-	+	3.0	32	1	800	TBSS	+	Screening	Eddy currents Motion	+	LLS
van Pul et al. 2012 [91]	-	-	+	3.0	32	1	800	Determ tract	+	Screening	Eddy currents Motion	+	
Vinall et al. 2013 [92]	+	+	-	1.5	12	1	600 700	ROI analysis	+			+	
Yoo et al. 2005 [93]	-	-	+	1.5	6	1	1,000	Determ tract	+	Screening		+	WLLS
Zwicker et al. 2013 [94]	-	-	-		12			Determ tract	+	Screening		+	WLLS

+ used, and – not used, *blank cells* indicate that details were not reported in the corresponding study, *Determ tract* deterministic tractography, *DTE* diffusion tensor estimation, *LLS* linear least squares, *Neo* neonatal, *NLLS* non-linear least squares, *No*. number, *Prob tract* probabilistic tractography, *Quality assess*. whether quality of neonatal DTI data was assessed, *Quality method* how quality assessment was performed, *ROI* region of interest, *TBSS* tract-based spatial statistics, *WLLS* weighted linear least squares, *Incub* incubator, *no. Dir* number of diffusion-encoding gradients, *no. B=0* number of non-diffusion weighted images, *Sed* Sedation, *Quality assess.* Quality assessment, *DTI excl.* Exclusion of poor quality datasets

### Dedicated neonatal MR imaging

Nineteen studies (26%) reported the use of dedicated neonatal scanning equipment; 19 (26%) papers reported the use of a neonatal head coil, which was installed in an MRI-compatible incubator in 18 (24%) of the cases. Sedative drugs were administered prior to scanning in 28 (38%) studies.

### Diffusion tensor imaging data acquisition parameters

Seventy-two studies (97%) reported the number of gradient directions at which diffusion tensor imaging (DTI) was performed; this number ranged from 6 to 44, with an average of 18 directions per scan. B values were reported in 71 studies (96%), and most were 600–1,000 s/mm^2^ (range 350–3,000, average 734 s/mm^2^, median 700 s/mm^2^). Number of non-diffusion-weighted images (*b* = 0) was reported in 57 (77%) studies, mostly limited to one or two *b* = 0 images per scan (range 1–16, average 1.47).

The static field strength of the MRI scanners was reported in 69 (93%) studies. The most frequently used MRI scanners were 1.5 tesla (*n* = 44, 60%), followed by 3 tesla (*n* = 25, 34%).

### Processing methods

Sixty-one studies (82%) reported quality assessment of the diffusion-weighted images. Forty-eight studies (65%) reported visual inspection of diffusion data, and three studies (4.1%) reported standardized software-driven quality assessment. Eleven studies (15%) reported quality assessment without further elaboration on how this was performed.

Fifty-six (76%) studies reported exclusion of datasets with insufficient quality. Specific correction methods were applied in 41 studies (55%); this was mostly restricted to correction for motion artefacts (*n* = 27, 37%) and eddy currents (*n* = 33, 45%). One study (1.4%) reported the use of automatic detection of outliers (corrupted slices as a result of artefacts or signal-loss) before tensor estimation [74].

Description of the diffusion tensor methodology was available in 56 papers (76%). Among studies that did describe tensor estimation methodology, linear least square and weighted linear least square were most frequently used (*n* = 29, 39% and *n* = 23, 31%, respectively).

Region-of-interest analysis was the most frequently used method of analysis (*n* = 37, 50%). Fiber tractography was applied in 25 studies (34%), of which 13 studies (18%) performed probabilistic tractography, 10 (14%) deterministic tractography and three (4.1%) did not describe which tractography approach was used. Voxel-wise analysis of diffusion data using tracts-based spatial statistics (TBSS) was performed in 15 studies (20.3%).

## Discussion

This systematic review demonstrates wide variation among preterm neonatal diffusion tensor imaging studies in hardware setup, acquisition parameters and post-processing settings. Many papers had an incomplete description of these matters.

### Acquisition settings

In most studies field strength, b-values and number of directions were reported, and both gradient strength and number of diffusion directions tended to increase over the years. However, reported acquisition parameters differed considerably among the studies. Even when evaluating settings for each year of publication separately, large differences among studies existed in the number of gradient directions and height of b values.

Usage of dedicated neonatal equipment such as specialized neonatal head coils and MRI-compatible incubators was only reported in a minority of studies. MRI-compatible incubators provide a safe and comfortable environment and might therefore reduce subject motion during acquisition. Because our results show that the majority of studies scan without using sedation, a comfortable environment is indispensable to keep the child comfortable and asleep during diffusion tensor imaging acquisition. Furthermore, using smaller head coils, adapted to the characteristics of the preterm brain, might result in higher signal-to-noise ratio [95, 96]. However, because signal-to-noise ratio depends on other features as well, it remains debatable whether dedicated neonatal head coils always provide the best signal-to-noise ratio. For further evaluation of benefits and limitations provided by specific neonatal equipment, it is important that research groups describe which scanning equipment was used and how this impacted scanning convenience and data quality.

### Quality assessment of diffusion tensor imaging data

For diffusion tensor imaging, it is known that even optimal equipment and acquisition parameters cannot guarantee appropriate data quality because diffusion tensor imaging is highly sensitive to artefacts. Frequent occurrence of motion during acquisition among preterm infants can result in signal dropout, misalignment of slices, and signal intensity inhomogeneity. In addition, the echo-planar imaging sequence frequently used in neonatal neuroimaging is susceptible to inhomogeneity at air–tissue boundaries [96]. Therefore well-informed processing steps to detect and correct image distortions properly are essential in neonatal diffusion tensor imaging. In a considerable number of studies, information regarding any kind of quality assessment was missing. When quality assessment was stated, detailed description of methodology was frequently not provided. Comprehensive information about precise visual inspection methodology is valuable because different visual inspection strategies might yield different results. Color maps, for example, can be very useful to identify corrupted data but often fail to display signal loss if it is limited to a small number of directions. Careful visual inspection of raw diffusion data in three orthogonal planes by an experienced observer seems to be more effective for this purpose [97]. Further software-based assessment of diffusion tensor imaging quality can reveal additional unobserved image distortions by pointing out more-dispersed signal loss and less-visible artefacts. Our results show that software-driven quality assessment is performed in a limited number of studies. Because there seems to be no consensus regarding assessment of diffusion tensor imaging data quality, a combined approach using multiple methods seems preferable. Such strategies are hardly reported in current neonatal diffusion tensor imaging literature.

When structured quality assessment is extensively performed, it is important to report this. For instance, because of the high likelihood of movement artefacts and signal loss of preterm brain diffusion tensor imaging data, it is often necessary to exclude diffusion data or even complete diffusion tensor imaging scans entirely from analysis to ensure reliability of results. Exclusion of datasets was not reported in 24.3% of the studies included in our review.

### Processing methodology

The influence of the chosen tensor estimation methodology on data quality is important to consider because different algorithms address outliers and errors differently [98, 99]. Appropriate algorithms for tensor estimation are crucial in premature infants because reliability of diffusion tensor imaging data depends on how corrupted slices or directions are dealt with. Our literature search showed that information regarding tensor algorithms was not provided in a considerable number of studies and that fast but less accurate tensor algorithms were most frequently used. Although more sophisticated tensor estimation algorithms have been developed and described, application of these methods in neonatal diffusion tensor imaging studies seems to be low. More robust tensor estimation methods that exclude motion-corrupted directions prior to computation of the diffusion tensor generally require more processing time but can result in significantly improved data quality [27, 99].

A large portion of studies in this review used advanced post-processing methods such as tractography and tract-based spatial statistics. Diffusion tensor imaging data quality is of special importance in these methods. Insufficient diffusion tensor quality can result in early abortion of tracking streamlines or aberrant tract propagation and might have serious effects on reliability of final results. Use of tract-based spatial statistics, accurate spatial co-registration of different datasets is only achievable when slices are perfectly aligned in every dataset. Misalignment of slices caused by head motion during scanning might result in erroneous co-registration, affecting the reliability of the results. Sophisticated correction for misalignment and exclusion of incorrigible datasets prior to co-registration are therefore essential.

### Future perspectives

**Fig. 2:**
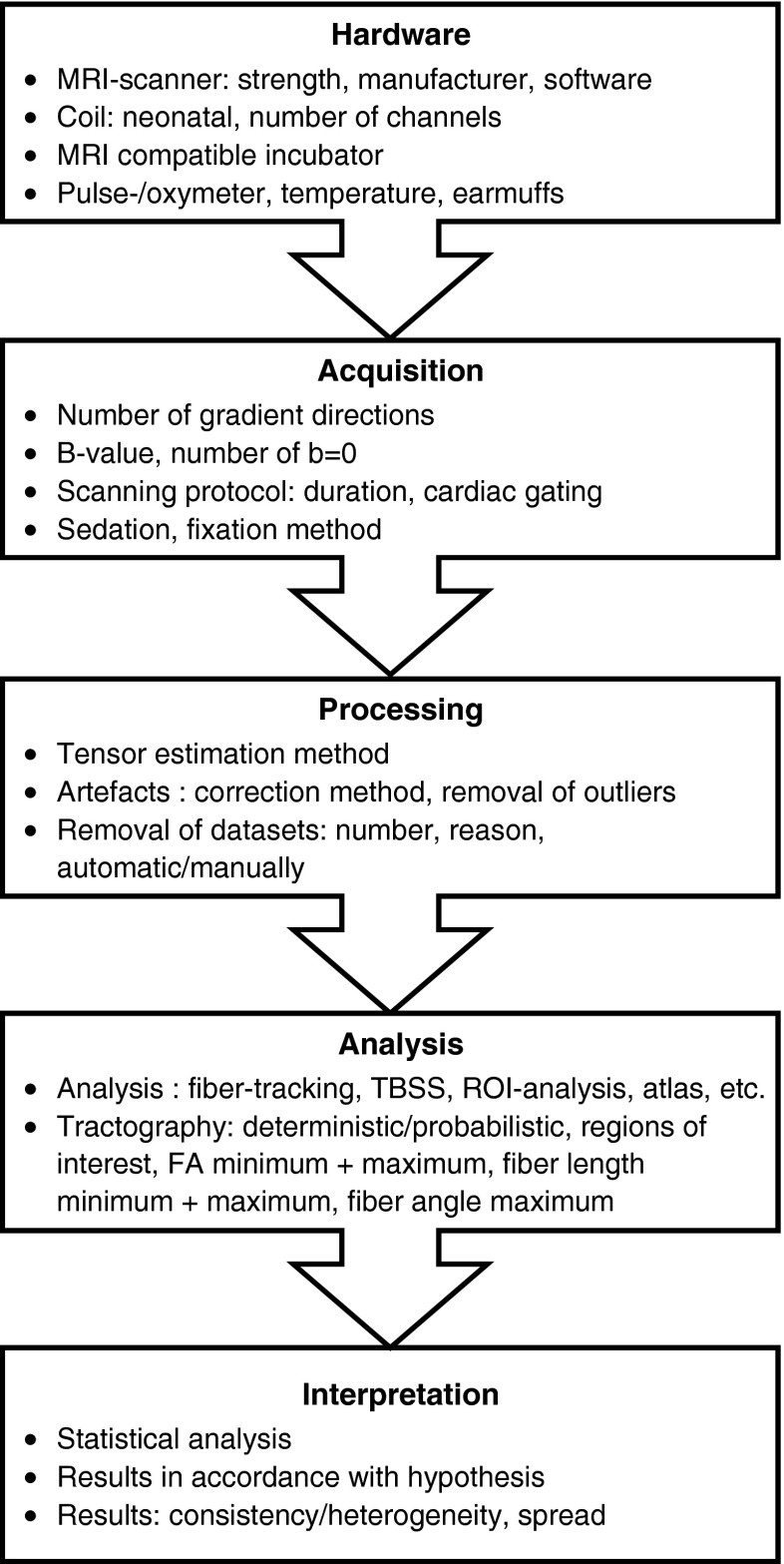
Overview of the processing pipeline for diffusion tensor imaging acquisition and analysis. Because all these steps determine data quality and analysis, reporting of these settings is valuable. Note: Outliers indicate motion-corrupted slices. *FA* fractional anisotropy, *ROI* regions of interest, *TBSS* tracts-based spatial statistics

Ideally, MRI workstations should be equipped with state-of-the-art quality-checking software, with direct feedback during image acquisition. Such on-the-flight correction allows immediate re-scanning of slices that contain artefacts. Further refinement of these techniques might lead to significant improvements in data quality. Development of even more sophisticated diffusion tensor imaging acquisition schemes, implementation of higher-order processing algorithms in neonatal neuroimaging and further development of user-friendly software to detect and correct poor-quality datasets can result in significant improvements in data quality [26]. Furthermore, providing samples of actual diffusion data as Electronic supplementary material would be very useful to allow the readers to assess image quality. Furthermore, because alterations in myelination, water content and synaptogenesis result in rapidly changing diffusion characteristics within the first year of life, population-specific, standardized acquisition settings and processing pipelines of neonatal diffusion data are urgently needed (Fig. 2) [100, 101].

## Conclusion

Diffusion tensor imaging has great potential for investigation of the preterm brain provided that acquisition and post-processing pipelines are adapted to its specific characteristics. Current clinical studies pay little attention to this methodological requirement. In order to make bigger steps forward in understanding preterm brain structure, development and injury mechanisms, maximal awareness of these matters is required.

## Electronic supplementary material

Below is the link to the electronic supplementary material.

ESM 1 (DOC 30 kb)
