# Supplementary material for: Data quality in diffusion tensor imaging studies of the preterm brain: a systematic review
Source: Pediatr Radiol. 2015 Mar 29;45(9):1372–81. doi: 10.1007/s00247-015-3307-y (PMC4526590; doi:10.1007/s00247-015-3307-y)
Supplement: Supplementary file 1 — (DOC 30 kb) [file 247_2015_3307_MOESM1_ESM.doc]

**Embase.com**

('diffusion tensor imaging'/de OR ('diffusion tensor' OR dti OR dtt):ab,ti) AND (brain/exp OR 'brain disease'/exp OR 'brain function'/exp OR 'brain development'/exp OR (cerebr* OR encephal* OR brain* OR 'corpus callosum' OR 'white matter' OR 'gray matter' OR cranial OR intracranial):ab,ti) AND (newborn/de OR prematurity/de OR 'gestational age'/de OR (newborn* OR (new* NEXT/1 born*) OR neonat* OR prematur* OR (pre NEXT/1 (matur* OR term*)) OR preterm* OR 'birth weight' OR gestation* OR postnatal):ab,ti) NOT ([animals]/lim NOT [humans]/lim)

**Medline OvidSP**

("diffusion tensor imaging"/ OR (diffusion tensor OR dti OR dtt).ab,ti.) AND (brain/ OR exp "brain diseases"/ OR (cerebr* OR encephal* OR brain* OR corpus callosum OR white matter OR gray matter OR cranial OR intracranial).ab,ti.) AND (exp "infant, newborn"/ OR "gestational age"/ OR (newborn* OR (new* ADJ born*) OR neonat* OR prematur* OR (pre ADJ (matur* OR term*)) OR preterm* OR "birth weight" OR gestation* OR postnatal).ab,ti.) NOT (exp animals/ NOT humans/)

**Cochrane central**

(('diffusion tensor' OR dti OR dtt):ab,ti) AND ((cerebr* OR encephal* OR brain* OR 'corpus callosum' OR 'white matter' OR 'gray matter' OR cranial OR intracranial):ab,ti) AND ((newborn* OR (new* NEXT/1 born*) OR neonat* OR prematur* OR (pre NEXT/1 (matur* OR term*)) OR preterm* OR 'birth weight' OR gestation* OR postnatal):ab,ti)

**Web-of-Science**

TS=(("diffusion tensor" OR dti OR dtt) AND (cerebr* OR encephal* OR brain* OR "corpus callosum" OR "white matter" OR "gray matter" OR cranial OR intracranial) AND (newborn* OR new born* OR neonat* OR prematur* OR pre matur* OR pre term* OR preterm* OR "birth weight" OR gestation* OR postnatal) NOT ((animal* OR mouse OR mice OR rat OR rats) NOT (human* OR patient*)))

**PubMed as supplied by publisher**

((diffusion tensor OR dti OR dtt)) AND ((cerebr*[tiab] OR encephal*[tiab] OR brain*[tiab] OR corpus callosum OR white matter OR gray matter OR cranial OR intracranial)) AND ((newborn*[tiab] OR new born*[tiab] OR neonat*[tiab] OR prematur*[tiab] OR pre matur*[tiab] OR pre term*[tiab] OR preterm*[tiab] OR birth weight OR gestation*[tiab] OR postnatal)) NOT medline[sb]

('diffusion tensor imaging'/de OR 'diffusion weighted imaging'/de OR ('diffusion tensor' OR 'diffusion weighted' OR dti OR dtt OR dwi):ab,ti) AND (brain/exp OR (cerebr* OR encephal* OR brain* OR 'corpus callosum' OR 'white matter' OR 'gray matter' OR cranial OR intracranial):ab,ti) AND (newborn/de OR prematurity/de OR (newborn* OR (new* NEXT/1 born*) OR neonat* OR prematur* OR (pre NEXT/1 (matur* OR term*)) OR preterm* OR 'birth weight' OR gestation*):ab,ti) NOT ([animals]/lim NOT [humans]/lim)
